# The plant alkaloid tetrandrine inhibits metastasis via autophagy-dependent Wnt/β-catenin and metastatic tumor antigen 1 signaling in human liver cancer cells

**DOI:** 10.1186/s13046-018-0678-6

**Published:** 2018-01-15

**Authors:** Zhenxing Zhang, Ting Liu, Man Yu, Kangdi Li, Wenhua Li

**Affiliations:** Hubei Key Laboratory of Cell Homeostasis, College of Life Sciences, Wuhan University, Wuhan, 430072 People’s Republic of China

**Keywords:** Autophagy, Metastasis, MTA1, Tetrandrine, Wnt/β-catenin, Epithelial-Mesenchymal transition (EMT)

## Abstract

**Background:**

Tetrandrine is a bisbenzylisoquinoline alkaloid isolated from the Chinese medicinal herb *Stephania tetrandra* S. Moore. We previously demonstrated that tetrandrine exhibits potent antitumor effects in many types of cancer cells. In this study, we investigated the effects of tetrandrine on human hepatocellular carcinoma (HCC) metastasis.

**Methods:**

The invasion and migration effects were evaluated via wound healing and transwell assays. Immunofluorescence and western blotting analyses were used to investigate the levels of epithelial-mesenchymal transition (EMT)-related protein. A metastasis model was established to investigate the inhibitory effect of tetrandrine on hepatocellular carcinoma metastasis in vivo.

**Results:**

Tetrandrine inhibits HCC invasion and migration by preventing cell EMT. The underlying mechanism was closely associated with tetrandrine-induced human liver cell autophagy, which inhibits Wnt/β-catenin pathway activity and decreases metastatic tumor antigen 1 (MTA1) expression to modulate cancer cell metastasis.

**Conclusion:**

Our findings demonstrate, for the first time, that tetrandrine plays a significant role in the inhibition of human hepatocellular carcinoma metastasis and provide novel insights into the application of tetrandrine in clinical HCC treatment.

**Electronic supplementary material:**

The online version of this article (10.1186/s13046-018-0678-6) contains supplementary material, which is available to authorized users.

## Background

Hepatocellular carcinoma (HCC) is one of the most common malignancies and results in approximately 600,000 deaths per year worldwide [1]. Although effective treatments, such as surgical resection, liver transplantation and percutaneous ablation, may be applied at earlier stages, the very poor prognosis and high recurrence of HCC after surgical resection result in a 5-year survival rate of less than 25% [2]. Therefore, intra-hepatic recurrence and the propensity for extrahepatic metastasis represent the major causes of death in the majority of HCC patients [2]. Modern chemotherapy regimens are used to treat these patients; however, there are underlying systemic risk factors [3]. In recent years, sorafenib was approved for the targeted treatment of advanced hepatocellular carcinoma [4].

Clinical data have indicated that sorafenib prolonged the median survival by approximately 3 months in patients with HCC [4]. With the common side effects of hand-foot skin reaction and diarrhea, sorafenib effectively controls primary tumor growth in early-stage HCC; however, it does not inhibit the development of secondary liver metastases or local and distant lymph node metastasis [5]. In addition, sorafenib has been reported to promote the invasiveness and metastasis of orthotopic HCC tumors in mice [6]. Therefore, effective treatments for advanced HCC are lacking, which poses a critical health problem. The development of an effective treatment, including therapeutic agents, for HCC patients in advanced stages with symptomatic progression is essential and urgently needed. In recent years, Chinese medicine has attracted interest for cancer therapeutics because of its comprehensive effect on cancer cells and low toxicity for normal cells.

Tetrandrine is a bisbenzylisoquinoline alkaloid isolated from the broadly used Chinese medicinal herb *Stephania tetrandra* S. Moore [7, 8]. Traditionally, tetrandrine has been used in China to treat patients with rheumatoid arthritis, hypertension, inflammation, sepsis and silicosis [8, 9]. Recently, numerous reports have indicated that tetrandrine is a promising chemotherapeutic agent with multiple anticancer effects [10–12]. Interestingly, we have shown that tetrandrine is a potent broad-spectrum agonist for cell autophagy in many types of cancer cells [13]. Autophagy is a catabolic process that involves protein and organelle degradation in the lysosome and the recycling of cellular components to ensure cellular survival during starvation under stress conditions [14, 15]. Anticancer agents, such as statins, inhibit tumor cell metastasis as a result of their ability to induce autophagy [16]. Thus, we speculate that tetrandrine may play a role in the regulation of cancer cell metastasis.

In the present study, we examined the efficacy of tetrandrine in HCC metastasis in vitro and in vivo. The results indicated that tetrandrine has the capacity to inhibit human liver cancer cell metastasis by preventing the epithelial-mesenchymal transition (EMT). In addition, the autophagy-dependent Wnt/β-catenin and MTA1 pathways are involved in tetrandrine-induced inhibition of metastasis. Thus, our findings suggest that tetrandrine treatment may have multiple beneficial effects as a potential treatment for HCC, and it not only affects the proliferation and survival of cancer cells but also suppresses tumor invasion and migration as an anti-metastasis agent.

## Methods

### Reagents and antibodies

Tetrandrine was purchased from Shanghai Ronghe Medical, Inc. (Shanghai, China) and dissolved at a concentration of 10 mM in DMSO as a stock solution. Recombinant human TGF-β1 was purchased from Peprotech (Peprotech Inc., USA) and used at the concentration of 5 ng·mL^− 1^. 3-Methyladenine (3-MA) and lithium chloride (LiCl) were purchased from Sigma-Aldrich (USA). Rabbit antibodies against E-cadherin, Vimentin, Occludin, MTA1, ATG7, CyclinD1, c-myc, β-catenin, phospho-β-catenin (Ser33/37/Thr41), GSK3β and phospho-GSK3β (Ser9) were purchased from Cell Signaling Technology (USA). The antibody against LC3 was obtained from Sigma. The antibodies against Wnt3a and GAPDH were purchased from Abcam (United Kingdom) and Beyotime (China).

### Cell lines and cell culture

The human hepatoma cell lines Huh7 and Hep3B were purchased from CCTCC (Wuhan, China) and cultured in Dulbecco’s modified Eagle’s medium (DMEM) containing 10% FBS. The cell line HCCLM9 was purchased from the Liver Cancer Institute (Fudan University, China) and cultured in RPMI 1640 media. The media were supplemented with 10% fetal bovine serum (FBS), 100 units·mL^− 1^ penicillin, and 100 μg·mL^− 1^ streptomycin. All cells were incubated at 37 °C in a humidified atmosphere of 5% CO_2_.

### Plasmid constructs and transfection

Human full-length MTA1 and Wnt3a were generated by PCR amplification of MTA1 and Wnt3a cDNA fragments. All cloned regions were verified by sequencing. For transient transfection, cells were transfected with the expression plasmids using FuGENE™ HD (Roche Applied Science, Switzerland) according to the manufacturer’s protocol. The stable transfection was performed as previously described [17].

### Total RNA extraction and quantitative real-time PCR

Total RNA was extracted using the Total RNA kit (OMEGA, USA) according to the manufacturer’s protocol. For this protocol, 1 μg of RNA was reverse transcribed into first strand cDNA using a RevertAid First Strand cDNA Synthesis Kit (Thermo Scientific, USA). Real-time PCR was performed using a System 7500 instrument (Applied Biosystems) with 2× FastStart Universal SYBR Green Master (Rox) (Roche Applied Science, USA) using the relative quantity (2^–ΔΔCt^) method. All reactions were performed in triplicate with internal duplicate determinations. The primer sequences were as follows: E-cadherin forward: 5’-GCCTCCTGAAAAGAGAGTGGAAG-3′, E-cadherin reverse: 5’-TGGCAGTGTCTCTCCAAATCCG-3′; Vimentin forward: 5′ -AGGCAAGCAGGAGTCCACTGA-3′, Vimentin reverse: 5′ -ATCTGGCGTTCCAGGGACTCAT -3′; MTA1 forward: 5′- CCAGGACCAAACCGCAGTAACA -3′, MTA1 reverse: 5′ -GTCAGCTTCGTCGTGTGCAGAT -3′; GAPDH forward: 5′- GTCTCCTCTGACTTCAACAGCG -3′, GAPDH reverse: 5′ -ACCACCCTGTTGCTGTAGCCAA -3′.

### CellTiter 96® AQueous one solution cell proliferation assay

Huh7, HCCLM9 and Hep3B cells were seeded in a 96-well plate at a cell density of 5 × 10^3^ cells/well. The cells were treated with the indicated concentrations (0-4 μM) of tetrandrine for 24 h. The cells were subsequently stained with 20 μL of MTS for 1-2 h, and the plates were read at 490 nm on a BioTek ELx800 (BioTek, US).

### Morphological examination

Huh7 or HCCLM9 cells were seeded in six-well culture dishes. The cells were subsequently serum-starved for approximately 10 h using D-hank’s balances (HyClone, USA). After treatment with 5 ng· mL^− 1^ TGF-β1 with or without tetrandrine for 72 h, the morphological changes of the cells were observed under an inverted phase-contrast microscope (Olympus, Japan). Photographs were obtained at 40X magnification using a Canon digital camera.

### Wound healing assay

Cells were seeded in 12-well culture dishes to grow and reached 80% confluence. The confluent monolayer was subsequently scratched in a straight line with a 200 μL sterile pipette tip. Floating cells were removed by careful washing with PBS prior to the addition of fresh culture medium with 2-μM tetrandrine or DMSO as the control. The process of wound healing was monitored using an inverted phase-contrast microscope (20X magnification) at the time of wounding and 24 h after scratching.

### Cell migration and invasion assay

For the transwell migration and invasion assays, 3 × 10^4^ and 6 × 10^4^ cells were seeded on the upper chamber of a transwell (8 μm pore size, BD Biosciences, NJ) or a Matrigel coated transwell (BD Biosciences, NJ) in serum-free media. The bottom chamber contained DMEM or RPMI 1640 media with 10% FBS and 2-μM tetrandrine or DMSO as a chemoattractant. The cells were fixed and stained with 0.1% crystal violet for 20 min at room temperature after incubation for 24 or 48 h. This step was followed by the gentle removal of the non-migrated or non-invaded cells from the upper chamber with a cotton swab. The number of cells that migrated or invaded was counted in 10 randomly selected fields (100X magnification).

### Anoikis assay

Anoikis was induced by culturing cells in agarose-coated plates as described by Frisch [18]. Briefly, the plates were pre-coated with 2 mL of 1% sterilized agarose. With agarose solidification, 2 × 10^5^ cells in 2% FBS medium were seeded onto the coated plates with DMSO or 2-μM tetrandrine for 72 h. The suspended cells were subsequently collected and subjected to apoptosis assays by Annexin V/PI staining on FACS.

### Preparation of cytoplasmic and nuclear extracts

Huh7 and HCCLM9 cells were treated with DMSO or 2-μM tetrandrine for 24 h, and the cytoplasmic and nuclear proteins were extracted using a Nuclear and Cytoplasmic Protein Extraction Kit according to the manufacturer supplied instructions (Beyotime Institute of Biotechnology, Jiangsu, China). The cytoplasmic and nuclear components were subsequently subjected to western bolt analysis.

### Western blot analysis

Western blotting was performed as previously described [19].

### Immunofluorescence (IF) staining

Huh7 and HCCLM9 cells were grown to 50% confluence on glass coverslips and treated with tetrandrine for the indicated times. The cells were subsequently fixed with 4% formaldehyde for 20 min. The fixed cells were then permeabilized in 0.5% TritonX-100 for 30 min. For F-actin staining, the fixed cells were stained with rhodamine-phalloidin (1:500) for approximately 45 min. For IF staining, the fixed cells were incubated with primary antibodies against E-cadherin, vimentin and β-catenin at 4 °C overnight after washing with PBS for 10 min. This procedure was followed by incubation with secondary antibody goat anti-rabbit FITC or goat anti-rabbit Cy3 for 1 h after washing with PBS. The cells were subsequently stained with DAPI (1:1000) for 10 min. E-cadherin, vimentin and β-catenin were detected using a confocal laser scanning microscope.

### CRISPR/Cas9 knockout (KO) system

ATG7-deficient Huh7 and HCCLM9 cells were established using the CRISPR/Cas9 system according to a standard protocol. pST1374-N-NLS-Flag-Linker-Cas9-BSD and pGL3-U6-gRNA-Puromycin mut Bsa1 ACCG vector were kindly provided by Xiaodong Zhang (Wuhan University, China). Briefly, sgRNA was generated and cloned into pGL3-U6-gRNA-Puromycin mut Bsa1 ACCG vector. Cas9 and sgRNA vectors were cotransfected into Huh7 and HCCLM9 cells for 24 h. Cells were subsequently selected by 2 μg·mL^− 1^ of puromycin and 20 μg·mL^− 1^ of blasticidin for an additional 24 h. Then, Huh7 and HCCLM9 cells were seeded into 96-well plates for monoclonalization. After approximately 3 weeks, ATG7 protein expression of the cell clones was detected by western blotting. Two pairs of small guide RNA (sgRNA) for ATG7 KO were used, and the primer sequences included 5’-ACCGAGAAGTACCACTTCTACTAT-3′ (forward) and 5′- AAACATAGTAGAAGTGGTACTTCT-3′ (reverse), 5’-ACCGTCCTTCTTAGAAGACTTGAC-3′ (forward) and 5’-AAACGTCAAGTCTTCTAAGAAGGA-3′(reverse).

### HCC xenograft tumor growth and lung metastasis model in vivo

Four-week-old male athymic BALB/c nu/nu SPF mice (body weight range from 18 g to 20 g) were obtained from the Hunan SJA Laboratory Animal Co., Ltd. (Changsha, Hunan, China). All animal care and experiments were approved by the Experimental Animal Center of Wuhan University. HCCLM9 WT and HCCLM9 ATG7 KO cells (5 million) resuspended in 0.2 mL of PBS were subcutaneously implanted into the right flank of each mouse. When the tumor volume reached approximately 50 mm^3^, the tumor-bearing mice were randomly divided into control and treatment groups (*n* = 6). The control and treatment groups were administered oral injection of vehicle (0.5% methylcellulose) and tetrandrine at 30 mg·kg^− 1^ of body weight every other day for 37 days. During the treatment, the tumor volumes were measured every day and were calculated using the following formula: V = 0.5 × large diameter×(small diameter)^2^. After the mice were sacrificed, the primary tumors were isolated for immunohistochemical staining and western blot analyses. The lungs were harvested and stained with hematoxylin and eosin to observe metastatic foci.

For the metastasis assay, HCCLM9 WT and HCCLM9 ATG7 KO cells were suspended in PBS, and 1.5 × 10^6^ cells (200 μL) were injected via the tail vein. Two days after the injection, treatment was initiated and orally administered every other day with 30 mg/kg tetrandrine or vehicle (0.5% methylcellulose). The mice were sacrificed at day 35 after injection, and the lungs were harvested and inspected for metastatic foci. The number of metastatic nodules in the lungs was counted.

### Statistical analysis

All data are presented as the mean ± standard deviation (SD) of at least three independent experiments. Student’s t tests were used for all statistical analyses, and *P* < 0.05 was considered statistically significant.

## Results

### Tetrandrine inhibits HCC cell invasion and migration

**Fig. 1:**
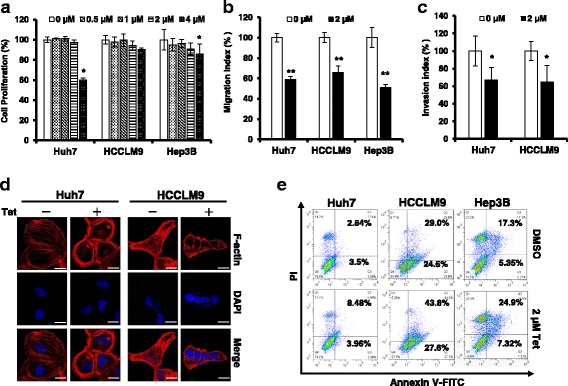
Tetrandrine inhibits HCC cell invasion and migration. **a** Data are presented as the mean ± SD of at least three independent experiments, *p < 0.05. **b** Transwell migration assay was conducted with Huh7, HCCLM9, and Hep3B cells treated with DMSO or 2-μM tetrandrine (Tet) for 24 h. The experiment was repeated at least three times, **p < 0.01. **c** The invasive properties of Huh7 and HCCLM9 cells treated with DMSO or 2-μM tetrandrine (Tet) for 48 h were assessed using a transwell invasion assay with a matrigel invasion chamber. The experiment was repeated three times, *p < 0.05. **d** Immunofluorescence staining for F-actin (using rhodamine phalloidin) in Huh7 and HCCLM9 cells after treatment with DMSO or 2-μM tetrandrine (Tet) for 24 h. Scale bars: 20 μm. **e** Anoikis assay. FACS analysis for anoikis after DMSO or 2-μM tetrandrine (Tet) treatment of Huh7, HCCLM9 and Hep3B cells for 72 h

To evaluate the effects of tetrandrine on HCC cells, Huh7, HCCLM9 and Hep3B cells were treated with 0 (DMSO), 0.5, 1, 2 or 4 μM of tetrandrine for 24 h. The cell proliferation assay indicated that tetrandrine exhibited almost no effect on the inhibition of HCC cell proliferation at 0.5-2 μM (Fig. 1a). However, tetrandrine inhibited HCC cell migration in a dose-dependent manner (Additional file 1: Figure S1A). Furthermore, a wound-healing and transwell assay showed that 2-μM tetrandrine significantly inhibited HCC cell migration and invasion (Fig. 1b, c and Additional file 1: Figure S1B, C and D). During tumor invasion and metastasis, as a result of changes in Rho-GTPase activity, the actin cytoskeleton will reorganize, which, in turn, causes the disruption of adherent junctions [20]. F-actin staining during this process indicated that 2-μM tetrandrine treatment resulted in the disappearance of parallel bundles of stress fibers (Fig. 1d). Anoikis induced by the disruption of the interactions surrounding the extracellular matrix was closely related to the metastasis potential of cells [21]. We subsequently examined the anoikis status after HCC cells were treated with tetrandrine. As shown in Fig. 1e, 2-μM tetrandrine treated cells underwent anoikis, which indicated that tetrandrine decreases cell survival when cells are blocked from inappropriate cell/extracellular matrix (ECM) interactions. Taken together, these results indicate that 2-μM tetrandrine could inhibit HCC cell invasion and migration.

**Fig. 2:**
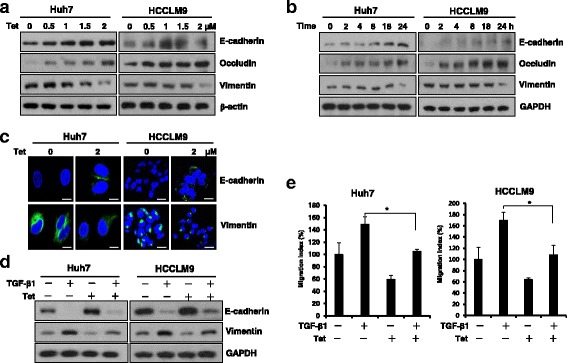
Tetrandrine prevented HCC cell EMT. **a** Western blot analysis of the EMT-related proteins E-cadherin, occludin and vimentin in Huh7 and HCCLM9 cells after treatment with the indicated concentrations (0-2 μM) of tetrandrine (Tet) for 24 h and (**b**) exposure to 2-μM tetrandrine for different time intervals (0-24 h); β-actin and GAPDH were used as the controls. **c** Huh7 and HCCLM9 cells were treated with 0 (DMSO) or 2-μM tetrandrine (Tet) for 24 h and then subjected to immunofluorescence analysis of the E-cadherin and vimentin levels. Scale bars: 20 μm. **d** Huh7 and HCCLM9 cells were treated with 2-μM tetrandrine (Tet) and TGF-β1 (5 ng·mL^− 1^) for 72 h; control cells were only treated with DMSO. EMT was examined by western blot analysis of the expression of related proteins, including E-cadherin and vimentin. GAPDH was loaded as a control. **e** Transwell migration assays were performed on Huh7 and HCCLM9 cells in the presence of 2-μM of tetrandrine (Tet) and 5 ng·mL^− 1^ of TGFβ-1 for the indicated time. Data are presented as the mean ± SD. of at least three independent experiments, *p < 0.05

### Tetrandrine prevented HCC cell EMT

As one of the crucial mechanisms that regulate the initial steps in metastatic progression, EMT is associated with cancer invasiveness and migration [22]. To examine whether the tetrandrine-inhibited cell migration and invasion were associated with the EMT, we initially measured the expression of epithelial and mesenchymal cell markers via real-time PCR, western blot and immunofluorescence staining. As shown in Fig. 2a–c and Additional file 1: Figure S2A, 2-μM tetrandrine exposure in Huh7 and HCCLM9 cells significantly increased the expression of the epithelial cell markers E-cadherin and occludin and decreased the level of the mesenchymal marker vimentin. Thus, we propose that tetrandrine-inhibited cell migration and invasion are associated with a block of EMT. To confirm this idea, we used transforming growth factor β1 (TGF-β1) to induce EMT of HCC cells, which has been well-characterized as an important inducer of EMT during carcinogenesis. When exposed to TGF-β1 for 72 h, Huh7 and HCCLM9 cells underwent EMT, in which cells change from an epithelial shape to a fibroblast-like shape. However, 2-μM tetrandrine rescued the TGF-β1-stimulated EMT by restoring cell morphology (Additional file 1: Figure S2B). Consistent with these morphological observations, the western blot and immunofluorescence analyses demonstrated that tetrandrine prevented the TGF-β1-induced progressive increase of vimentin and decrease of E-cadherin (Fig. 2d and Additional file 1: Figure S2C). In addition, as expected, a wound-healing and transwell assay indicated that 2-μM tetrandrine inhibited TGF-β1-induced cell migration (Fig. 2e and Additional file 1: Figure S2D, E). Thus, we demonstrated that tetrandrine-induced inhibition of HCC cell migration is associated with blocking of cell EMT.

### Tetrandrine-inhibited HCC cell migration is associated with autophagy

**Fig. 3:**
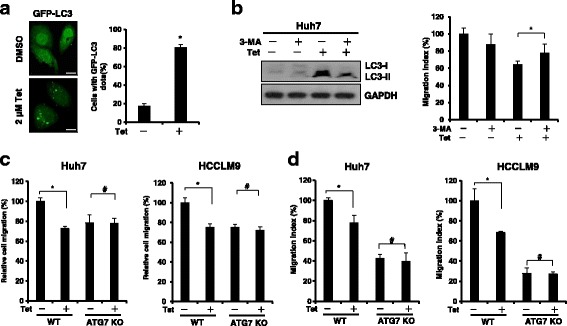
Tetrandrine-inhibited HCC cell migration is associated with autophagy. **a** Huh7 cells were transiently transfected with the GFP-LC3 plasmid for 12 h and subsequently treated with DMSO or 2- μM tetrandrine for 24 h. Immunofluorescence analysis of the cellular localization patterns of the GFP-LC3 fusion protein. Scale bars: 20 μm. The percentage of cells with GFP-LC3 puncta was quantified. Data are represented as the mean ± SD. *p < 0.05. **b** Huh7 cells were pretreated with 3-MA (3 mM) for 1 h and then treated with DMSO or 2-μM tetrandrine for 24 h. Western blot analysis of LC3 levels. GAPDH was loaded as a control. Transwell migration assays were performed on Huh7 cells in the presence of 2-μM tetrandrine (Tet) and/ or 3-MA for 24 h. Data are presented as the mean ± SD. of at least three independent experiments, *p < 0.05. **c** A would healing and (**d**) a transwell migration assay was conducted for WT and ATG7-deficient Huh7 and HCCLM9 cells treated with DMSO or 2-μM tetrandrine (Tet) for 24 h. Data are presented as the mean ± SD. of at least three independent experiments, #*p* > 0.01, *p < 0.05

Autophagy is a double-edged sword in tumorigenesis, metastasis and anticancer therapies [23]. Many antitumor agents have the ability to inhibit cell migration through the induction of cancer cell autophagy [16]. We have previously reported that tetrandrine is a potent broad-spectrum agonist for cell autophagy [13]. Thus, we determined whether tetrandrine-induced inhibition of HCC cell migration was related to autophagy. As shown in Fig. 3a, 2-μM tetrandrine significantly evoked the expression of GFP-LC3 fluorescent puncta of Huh7 cells. Autophagosome-lysosome fusion was induced when the treatment combined tetrandrine and chloroquine (CQ)/Bafilomycin (BafA1) (Additional file 1: Figure S3A). In addition, 3-methyladenine (3-MA), a common specific inhibitor of autophagy, significantly blocked the expression of LC3 and rescued cell migration inhibition (Fig. 3b and Additional file 1: Figure S3B). These results indicate that the tetrandrine-induced inhibition of Huh7 cell migration is likely linked to the induction of autophagy. To further confirm this idea, we constructed an ATG7 knockout cell line using the CRISPR/Cas9 system in Huh7 and HCCLM9 cells, which displayed autophagy defects in response to treatment with tetrandrine (Additional file 1: Figure S3C). Would healing and transwell migration results indicated that tetrandrine did not inhibit ATG7-knockout cell migration (Fig. 3c, d and Additional file 1: Figure S3D, E). Thus, the suppression of autophagy abrogated tetrandrine-induced inhibition of cell migration. Based on these results, we concluded that tetrandrine-induced inhibition of HCC cell migration is associated with the induction of autophagy.

### Autophagy-dependent Wnt/β-catenin signaling is involved in tetrandrine-induced inhibition of HCC cell migration

**Fig. 4:**
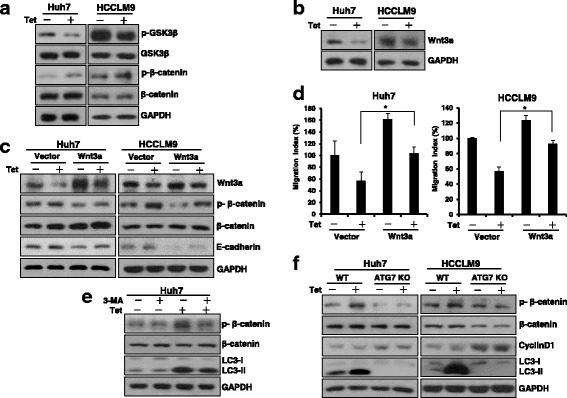
Autophagy-dependent Wnt/β-catenin pathway was involved in tetrandrine-inhibition of HCC cell migration. **a** Western blot analysis of p-GSK3β, total GSK3β, p-β-catenin, and total β-catenin protein levels after Huh7 and HCCLM9 cells were treated with DMSO or 2-μM tetrandrine (Tet) for 24 h. **b** Tetrandrine (Tet) decreases Wnt3a protein expression. **c** Overexpression of Wnt3a in Huh7 and HCCLM9 cells that were treated with DMSO or 2-μM tetrandrine (Tet) for 24 h. Wnt3a, p-β-catenin, total β-catenin and E-cadherin protein expressions were determined by western blot. **d** A transwell migration assay was performed on empty vector and Wnt3a-overexpression Huh7 and HCCLM9 cells that were treated with DMSO or 2-μM tetrandrine (Tet) for 24 h. The experiment was repeated three times, *p < 0.05. **e** Tetrandrine-inhibited Wnt/β-catenin activation was rescued by an autophagy inhibitor. Huh7 cells were pretreated with 3-MA (3 mM) for 1 h, followed by treatment with DMSO or 2-μM tetrandrine for 24 h; cell lysates were subjected to western blot analysis to detect the p-β-catenin and total β-catenin expression. **f** WT and ATG7-deficient Huh7 and HCCLM9 cells were treated with or without 2-μM tetrandrine for 24 h, followed by western blot analysis of p-β-catenin, total β-catenin, CyclinD1 and LC3 expression. GAPDH was loaded as an internal control

Numerous reports have shown that Wnt/β-catenin signaling is crucial in tumor metastasis and normal development [24, 25]. Thus, we subsequently examined whether the tetrandrine-inhibited HCC cell migration is associated with the Wnt/β-catenin pathway. Western blot analysis showed that in addition to p-GSK3β down-regulation, tetrandrine increased the p-β-catenin protein level, which implied that tetrandrine repressed Wnt/β-catenin signaling activity (Fig. 4a). Furthermore, nuclear extracts and immunofluorescence analysis showed that tetrandrine increased the accumulation of β-catenin in the cytoplasm and inhibited β-catenin translocation to the nucleus (Additional file 1: Figure S4A and B). Moreover, β-catenin transcriptional activity was inhibited by tetrandrine (Additional file 1: Figure S4C). LiCl is an activator of the Wnt/β-catenin pathway through the upregulation of phosphorylated-GSK3β. Tetrandrine also inhibited LiCl-induced Wnt/β-catenin activity (Additional file 1: Figure S4D). Tetrandrine substantially decreased Wnt3a protein levels (Fig. 4b). Moreover, Wnt3a degradation was dependent on tetrandrine induced autophagy (Additional file 1: Figure S4E). To determine the role of Wnt3a in tetrandrine-inhibited HCC cell EMT and metastasis, Huh7 and HCCLM9 cells were stably transfected with a Wnt3a vector. As shown in Fig. 4c and d and Additional file 1: Figure S4F and G, in addition to promoting Wnt pathway activation, Wnt3a overexpression markedly rescued the inhibition of EMT and migration of HCC cells in the presence of tetrandrine treatment. However, Wnt3a overexpression was not significantly different in the promotion of cell migration in ATG7 KO Huh7 cells compared with vector cells (Additional file 1: Figure S4H). Evidence indicates that autophagy modulates various cellular signals, including Wnt/β-catenin pathways [26]. Thus, we subsequently examined whether a relationship exists between Wnt/β-catenin signaling and autophagy as a result of tetrandrine treatment. As shown in Fig. 4e, with the block of autophagy, 3-MA distinctly impaired the tetrandrine effect of inhibiting β-catenin activation. In contrast, overexpression of Wnt3a activated β-catenin and upregulated downstream cyclinD1 and c-myc levels; however, it did not inhibit tetrandrine-induced autophagy (Additional file 1: Figure S4I). Tetrandrine did not induce autophagy of ATG7-knockout HCC cells, and the reduced expression of p-β-catenin and increased expression of CyclinD1 were also not inhibited by tetrandrine (Fig. 4f). These data suggest that tetrandrine inhibition of HCC cell migration occurs via the suppression of the Wnt/β-catenin pathway, which is regulated by tetrandrine-induced autophagy.

### Tetrandrine inhibits HCC migration, in part through MTA1

**Fig. 5:**
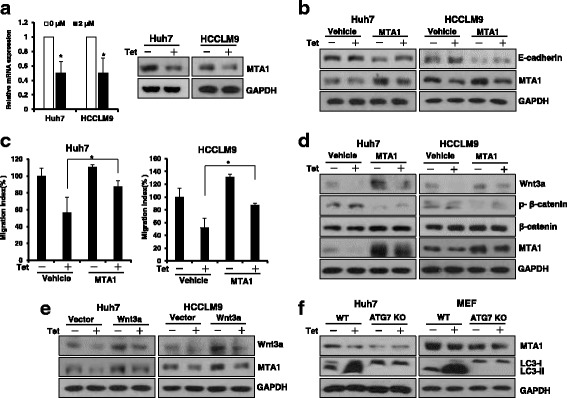
Tetrandrine inhibited HCC migration, in part through MTA1. **a** Expression analysis of MTA1 by quantitative real time-PCR and western blotting 24 h after treatment of Huh7 and HCCLM9 cells with or without 2-μM tetrandrine. Data are presented as the mean ± SD. of at least three independent experiments, *p < 0.05. **b** Huh7 and HCCLM9 cells were transfected with empty vector or MTA1 expression plasmid for 48 h. Cells were subsequently treated with DMSO or 2-μM tetrandrine (Tet) for 24 h, followed by western blot analysis of MTA1 and E-cadherin expression. GAPDH was loaded as an internal control. **c** A transwell migration assay demonstrated that MTA1 overexpression rescued tetrandrine-induced migration inhibition of Huh7 and HCCLM9 cells. *p < 0.05. **d** Huh7 and HCCLM9 cells were transfected with emptor vector or MTA1 overexpression plasmid for 48 h and then treated with DMSO or 2-μM tetrandrine for 24 h. Wnt signal related proteins, including Wnt3a, p-β-catenin, total β-catenin expression, and MTA1 levels, were detected by western blotting. **e** MTA1 expression was determined by western blotting after Wnt3a-overexpressed Huh7 and HCCLM9 cells were treated with or without 2-μM tetrandrine for 24 h. **f** Western blotting to detect MTA1 and LC3 expression after WT and ATG7-deficient Huh7 and MEF cells were treated with DMSO or 2-μM tetrandrine for 24 h

Metastatic tumor antigen 1 (MTA1) is correlated with cancer aggressiveness and an increase in the migration and invasion of various human tumors by modulating the expression of target genes [27]. We examined whether MTA1 was involved in tetrandrine-induced inhibition of HCC cell migration. As shown in Fig. 5a, tetrandrine treatment substantially decreased MTA1 expression at both the mRNA and protein levels. However, overexpression of MTA1 partially restored E-cadherin levels and rescued tetrandrine-inhibited migration of Huh7 and HCCLM9 cells (Fig. 5b, c and Additional file 1: Figure S5A, B, C). The overexpression of MTA1 in ATG7 knockout Huh7 cells promoted cell migration (Additional file 1: Figure S5D). Evidence has indicated that MTA1 was closely correlated with the Wnt signaling components [28]. Thus, we subsequently examined β-catenin activity in the presence of MTA1 overexpression. As shown in Fig. 5d, MTA1 increased Wnt3a and decreased phosphorylated β-catenin levels, which is suggestive of the activation of Wnt signaling. Correspondingly, upregulation of Wnt3a also promoted MTA1 protein levels regardless of tetrandrine treatment (Fig. 5e). These results indicated that MTA1 expression and Wnt/β-catenin signaling are mutually regulated by each other. Finally, we considered whether tetrandrine-regulated MTA1 expression was related to autophagy. Additional file 1: Figure S5E shows that the inhibition of autophagy by 3-MA resulted in an increase in MTA1 protein levels in the presence of tetrandrine. Moreover, tetrandrine did not reduce MTA1 expression in autophagy-deficient ATG7 knockout HCC cells (Fig. 5f). Therefore, these results demonstrated that tetrandrine inhibited HCC migration, at least in part, through autophagy degraded MTA1.

### Tetrandrine inhibits HCC metastasis in vivo

**Fig. 6:**
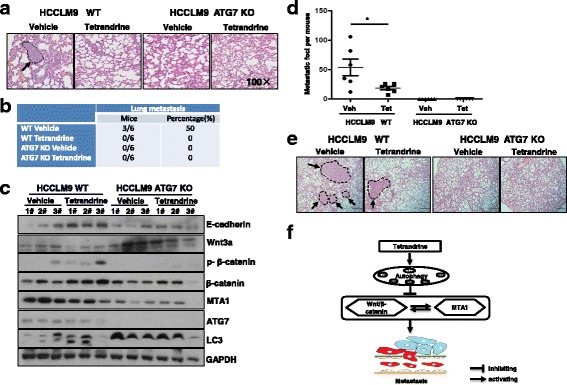
Tetrandrine inhibits HCC cell metastasis in vivo. **a** HE staining of metastatic tumors in the lung of WT HCCLM9 or ATG7-deficient HCCLM9 cell xenografts treated with vehicle (0.5% methylcellulose) or 30 mg·kg^− 1^ tetrandrine as indicated by arrows. Magnification is 100X. **b** Incidence of lung metastasis in pulmonary metastasis model with WT and ATG7-deficient HCCLM9 cells. **c** Western blot analysis of protein lysis isolated from 30 mg·kg^− 1^ tetrandrine or vehicle treated WT and ATG7-deficient HCCLM9 cell xenografts. **d** Incidence of lung metastasis in tail vein injection metastasis model with WT and ATG7-deficient HCCLM9 cells. *p < 0.05. **e** HE staining of metastatic nodules in the lung of WT HCCLM9 or ATG7-deficient HCCLM9 cells. Metastatic foci are indicated by arrows. Scale bars: 50 μm. **f** A putative model in which tetrandrine inhibits HCC metastasis by repression of multiple target genes

To evaluate the effect of tetrandrine on the inhibition of tumor metastasis in vivo, we initially established HCCLM9 subcutaneous tumor xenograft models with athymic nude mice. When the tumor volume reached approximately 50 mm^3^, nude mice were orally administered vehicle or tetrandrine (30 mg·kg^− 1^) every other day for 37 days. As shown in Additional file 1: Figure S6A, tetrandrine treatment inhibited tumor growth by reducing the tumor volume and weight. Importantly, we determined that treatment with 30 mg·kg^− 1^ tetrandrine for 5 weeks inhibited HCCLM9 cell lung metastasis in vivo (Fig. 6a and b). In contrast, tetrandrine did not inhibit the growth of tumors that originated from ATG7-knockout HCCLM9 cells with an autophagy defect (Additional file 1: Figure S6B). These results were consistent with our previous report that the inhibition of tumor growth by tetrandrine is related to autophagy in vivo [29]. More interestingly, lung metastasis was not observed in mice with ATG7-knockout HCCLM9 cell tumors regardless of tetrandrine treatment (Fig. 6a and b). Thus, we demonstrated that tetrandrine-induced inhibition of HCC tumor metastasis in vivo is associated with autophagy. Furthermore, the detection of LC3 protein levels in the tumor tissue samples also verified that tumors from ATG7-knockout HCCLM9 cells did not undergo autophagy even when treated with tetrandrine (Fig. 6c). Moreover, western blot and immunohistochemistry analyses indicated that tetrandrine inhibited EMT and reduced Wnt/β-catenin and MTA1 activity in vivo (Fig. 6c and Additional file 1: Figure S6C). Collectively, these results suggest that, consistent with the in vitro results, tetrandrine inhibits HCC metastasis in vivo by modulating cell EMT and the Wnt/β-catenin and MTA1 signaling pathways.

To further demonstrate the effect of tetrandrine on the inhibition of HCC metastasis in vivo, HCCLM9 WT and HCCLM9 ATG7 KO cells were injected via the tail vein. Vehicle or tetrandrine (30 mg·kg^− 1^) were administered every other day for 35 days. We determined that tetrandrine treatment substantially inhibited HCCLM9 WT cell lung metastasis. Surprisingly, lung metastasis was not observed in the mice with ATG7-knockout HCCLM9 cells, regardless of tetrandrine treatment (Fig. 6d, e and Additional file 1: Figure S6D), which was consistent with the xenograft model results. Moreover, no body weight loss was observed after tetrandrine treatment (Additional file 1: Figure S6E).

## Discussion

Tetrandrine, as a traditional Chinese medicine, has recently attracted considerable attention for its potential to treat various diseases, including heart and cerebrovascular diseases, inflammatory-related syndromes, Ebola virus, and cancer [8, 9, 30]. Numerous reports have suggested that tetrandrine may be used as a cancer chemotherapeutic agent for many types of cancers because of its potent antitumor effects [10, 11]. We have previously demonstrated that tetrandrine effectively induces apoptosis at high concentrations and stimulates autophagy at low concentrations in human HCC cells [29, 31]. Furthermore, tetrandrine induces differentiation in leukemia cells [19]. Moreover, tetrandrine shows good synergistic antitumor effects in combination with other chemotherapy agents [32, 33]. Here, we demonstrated that tetrandrine inhibits metastasis in human hepatocellular carcinoma through an autophagy-dependent pathway. Therefore, tetrandrine is a promising anti-cancer drug with diverse benefits in cancer therapy. Our previous studies have shown that one of the critical actions of tetrandrine on cancer cells is its ability to induce cancer cell autophagy.

Autophagy is important for maintaining intercellular homeostasis, and it serves as a temporary survival mechanism [34, 35]. Autophagy possesses numerous connections to various human diseases and stressors, as well as normal developmental processes and aging [36]. For cancer therapy, in general, the roles of autophagy are complicated, with two primary and opposing functions (cytoprotective and cytotoxic) in tumor cells in response to stress induced by chemotherapy or radiation [37]. As a cytoprotective function, autophagy may facilitate the migration of tumor cells by protecting cells from death during different metastasis processes. In contrast, autophagic cell death also acts as a strong barrier against cancer progression by eliminating cancer cells that may detach from the primary site and intravasate into the vasculature [37]. In the current study, tetrandrine substantially induced anoikis and removed seed cells with metastasis potential (Fig. 1e). Thus, we demonstrated that tetrandrine has a cytotoxic function in the inhibition of the metastasis of human hepatocellular carcinoma. In addition, tetrandrine inhibited HCC cell invasion and migration by repressing autophagy-dependent Wnt/β-catenin and MTA1 signaling (Fig. 6f). In this respect, tetrandrine acted as a potent autophagy agonist and was a crucial agent for the regulation of gene activity involved in tumor metastasis in an autophagy-modulated mechanism. Although autophagy may be a critical process that promotes the degradation of major proteins, such as snail and twist, which regulate EMT, the role of degradation in tetrandrine-induced inhibition of HCC metastasis must be clarified in the present research model.

Of note, tetrandrine inhibited Wnt/β-catenin pathway activity, which significantly contributes to the regulation of the metastatic progression of cancer. Although the underlying mechanisms are not entirely clear, the activation of Wnt/β-catenin signaling in cancer often drives a transcriptional program that may enhance cell migration and invasiveness by promoting the expression of MMPs and initiating EMT [24, 25]. Thus, the identification of small molecules that target specific Wnt signaling components may be therapeutically useful. In this study, we determined that tetrandrine-suppressed Wnt/β-catenin pathway activity is associated with an impairment of HCC cell migration. Natural compounds, such as curcumin and quercetin, have also been shown to have anticancer activities through the attenuation of Wnt signaling [38, 39]. In addition, the inhibition of Wnt signals may increase cell sensitivity to chemotherapeutic agents in various cancer cells, which may be advantageous in the development of combinatorial therapies. We have also previously demonstrated that tetrandrine exhibited synergistic antitumor effects in combination with sorafenib or chloroquine; however, the effects of tetrandrine on the suppression of the Wnt/β-catenin pathway in this process must be further evaluated.

MTA is another critical regulatory molecule that is closely related to transformation and tumor progression, which increases migration and invasion by modulating the expression of target genes [27]. MTA1 has been shown to regulate the response of oncogenes and tumor suppressor genes [27]. In this study, our data indicated that tetrandrine inhibited HCC migration, in part by decreasing MTA1 expression at the transcriptional level. The underlying mechanism involved in tetrandrine suppression of cell EMT is the regulation of MTA1 expression. Several studies have shown that MTA1 acts as an important upstream modifier of Wnt1 signaling in cancer cells [28]. The current findings demonstrated that MTA1 and Wnt3a can promote the expression of each other (Fig. 5).

## Conclusions

Collectively, our previous and current results demonstrate that tetrandrine plays a significant role in inhibiting human hepatocellular carcinoma metastasis both in vitro and in vivo. The potential molecular mechanisms of tetrandrine involve the modulation of autophagy-dependent Wnt/β-catenin and MTA1 signaling. Therefore, our findings provide novel insights into the application of tetrandrine in clinical HCC therapies and suggest that Chinese herbal medicine has broad prospects in the treatment of human cancer.

## Additional file

Additional file 1: Supplementary figures and figure legends. (DOCX 3775 kb)

## Abbreviations

3-MA: 3-methyladenine
ATG7: Autophagy associated gene 7
DMSO: Dimethyl sulfoxide
EMT: Epithelial-mesenchymal transition
HCC: Hepatocellular carcinoma
LC3: Microtubule-associated protein 1A/1B-light chain 3
MTA1: Metastatic tumor antigen 1
Tet: Tetrandrine
TGF-β1: Transforming growth factor beta 1

## References

[1] Llovet JM, Burroughs A, Bruix J (2003). Hepatocellular carcinoma. Lancet.

[2] Forner A, Llovet JM, Bruix J (2012). Hepatocellular carcinoma. Lancet.

[3] Llovet JM, Bruix J (2008). Novel advancements in the management of hepatocellular carcinoma in 2008. J Hepatol.

[4] Kane RC, Farrell AT, Madabushi R, Booth B, Chattopadhyay S, Sridhara R, Justice R, Pazdur R (2009). Sorafenib for the treatment of unresectable hepatocellular carcinoma. Oncologist.

[5] Llovet JM, Ricci S, Mazzaferro V, Hilgard P, Gane E, Blanc JF, de Oliveira AC, Santoro A, Raoul JL, Forner A (2008). Sorafenib in advanced hepatocellular carcinoma. N Engl J Med.

[6] Zhang W, Sun HC, Wang WQ, Zhang QB, Zhuang PY, Xiong YQ, Zhu XD, Xu HX, Kong LQ, Wu WZ (2012). Sorafenib down-regulates expression of HTATIP2 to promote invasiveness and metastasis of orthotopic hepatocellular carcinoma tumors in mice. Gastroenterology.

[7] Liu L, Cao Y, Chen C, Zhang X, Mc Nabola A, Wilkie D, Wilhelm S, Lynch M, Carter C (2006). Sorafenib blocks the RAF/MEK/ERK pathway, inhibits tumor angiogenesis, and induces tumor cell apoptosis in hepatocellular carcinoma model PLC/PRF/5. Cancer Res.

[8] Wu JM, Chen Y, Chen JC, Lin TY, Tseng SH (2010). Tetrandrine induces apoptosis and growth suppression of colon cancer cells in mice. Cancer Lett.

[9] Shen DF, Tang QZ, Yan L, Zhang Y, Zhu LH, Wang L, Liu C, Bian ZY, Li H (2010). Tetrandrine blocks cardiac hypertrophy by disrupting reactive oxygen species-dependent ERK1/2 signalling. Br J Pharmacol.

[10] He BC, Gao JL, Zhang BQ, Luo Q, Shi Q, Kim SH, Huang E, Gao Y, Yang K, Wagner ER (2011). Tetrandrine inhibits Wnt/beta-catenin signaling and suppresses tumor growth of human colorectal cancer. Mol Pharmacol.

[11] Qin R, Shen H, Cao Y, Fang Y, Li H, Chen Q, Xu W (2013). Tetrandrine induces mitochondria-mediated apoptosis in human gastric cancer BGC-823 cells. PLoS One.

[12] Liu T, Liu X, Li W (2016). Tetrandrine, a Chinese plant-derived alkaloid, is a potential candidate for cancer chemotherapy. Oncotarget.

[13] Wang H, Liu T, Li L, Wang Q, Yu C, Liu X, Li W (2015). Tetrandrine is a potent cell autophagy agonist via activated intracellular reactive oxygen species. Cell Biosci.

[14] Levine B (2007). Cell biology: autophagy and cancer. Nature.

[15] Klionsky DJ, Emr SD (2000). Autophagy as a regulated pathway of cellular degradation. Science.

[16] Zhang J, Yang Z, Xie L, Xu L, Xu D, Liu X (2013). Statins, autophagy and cancer metastasis. Int J Biochem Cell Biol.

[17] Gong K, Xie J, Yi H, Li W (2012). CS055 (Chidamide/HBI-8000), a novel histone deacetylase inhibitor, induces G1 arrest, ROS-dependent apoptosis and differentiation in human leukaemia cells. Biochem J.

[18] Frisch SM, Francis H (1994). Disruption of epithelial cell-matrix interactions induces apoptosis. J Cell Biol.

[19] Liu T, Men Q, Wu G, Yu C, Huang Z, Liu X, Li W (2015). Tetrandrine induces autophagy and differentiation by activating ROS and Notch1 signaling in leukemia cells. Oncotarget.

[20] Wang J, Wu Q, Zhang LH, Zhao YX, Wu X. The role of RhoA in vulvar squamous cell carcinoma: a carcinogenesis, progression, and target therapy marker. Tumour Biol. 2016;37(3):2879–90. 10.1007/s13277-015-4087-6 26409448

[21] Liotta LA, Kohn E (2004). Anoikis: cancer and the homeless cell. Nature.

[22] Lamouille S, Connolly E, Smyth JW, Akhurst RJ, Derynck R (2012). TGF-beta-induced activation of mTOR complex 2 drives epithelial-mesenchymal transition and cell invasion. J Cell Sci.

[23] White E, RS DP (2009). The double-edged sword of autophagy modulation in cancer. Clin Cancer Res.

[24] Qi J, Yu Y, Akilli Ozturk O, Holland JD, Besser D, Fritzmann J, Wulf-Goldenberg A, Eckert K, Fichtner I, Birchmeier W. New Wnt/beta-catenin target genes promote experimental metastasis and migration of colorectal cancer cells through different signals. Gut. 2016;65(10):1690–701. 10.1136/gutjnl-2014-307900 26156959

[25] Yang X, Li L, Huang Q, Xu W, Cai X, Zhang J, Yan W, Song D, Liu T, Zhou W (2015). Wnt signaling through Snail1 and Zeb1 regulates bone metastasis in lung cancer. Am J Cancer Res.

[26] Gao C, Cao W, Bao L, Zuo W, Xie G, Cai T, Fu W, Zhang J, Wu W, Zhang X (2010). Autophagy negatively regulates Wnt signalling by promoting Dishevelled degradation. Nat Cell Biol.

[27] Sen N, Gui B, Kumar R (2014). Role of MTA1 in cancer progression and metastasis. Cancer Metastasis Rev.

[28] Kumar R, Balasenthil S, Manavathi B, Rayala SK, Pakala SB (2010). Metastasis-associated protein 1 and its short form variant stimulates Wnt1 transcription through promoting its derepression from Six3 corepressor. Cancer Res.

[29] Gong K, Chen C, Zhan Y, Chen Y, Huang Z, Li W (2012). Autophagy-related gene 7 (ATG7) and reactive oxygen species/extracellular signal-regulated kinase regulate tetrandrine-induced autophagy in human hepatocellular carcinoma. J Biol Chem.

[30] Sakurai Y, Kolokoltsov AA, Chen CC, Tidwell MW, Bauta WE, Klugbauer N, Grimm C, Wahl-Schott C, Biel M, Davey RA (2015). Ebola virus. Two-pore channels control Ebola virus host cell entry and are drug targets for disease treatment. Science.

[31] Liu C, Gong K, Mao X, Li W (2011). Tetrandrine induces apoptosis by activating reactive oxygen species and repressing Akt activity in human hepatocellular carcinoma. Int J Cancer.

[32] Wan J, Liu T, Mei L, Li J, Gong K, Yu C, Li W (2013). Synergistic antitumour activity of sorafenib in combination with tetrandrine is mediated by reactive oxygen species (ROS)/Akt signaling. Br J Cancer.

[33] Mei L, Chen Y, Wang Z, Wang J, Wan J, Yu C, Liu X, Li W (2015). Synergistic anti-tumour effects of tetrandrine and chloroquine combination therapy in human cancer: a potential antagonistic role for p21. Br J Pharmacol.

[34] Marino G, Salvador-Montoliu N, Fueyo A, Knecht E, Mizushima N, Lopez-Otin C (2007). Tissue-specific autophagy alterations and increased tumorigenesis in mice deficient in Atg4C/autophagin-3. J Biol Chem.

[35] Luo M, Zhao X, Song Y, Cheng H, Zhou R (2016). Nuclear autophagy: an evolutionarily conserved mechanism of nuclear degradation in the cytoplasm. Autophagy.

[36] Mathew R, White E (2011). Autophagy in tumorigenesis and energy metabolism: friend by day, foe by night. Curr Opin Genet Dev.

[37] Kenific CM, Thorburn A, Debnath J (2010). Autophagy and metastasis: another double-edged sword. Curr Opin Cell Biol.

[38] Shan BE, Wang MX, Li RQ (2009). Quercetin inhibit human SW480 colon cancer growth in association with inhibition of cyclin D1 and survivin expression through Wnt/beta-catenin signaling pathway. Cancer Investig.

[39] Chen QY, Jiao DM, Wang LF, Wang L, Hu HZ, Song J, Yan J, Wu LJ, Shi JG (2015). Curcumin inhibits proliferation-migration of NSCLC by steering crosstalk between a Wnt signaling pathway and an adherens junction via EGR-1. Mol BioSyst.

