# Supplementary material for: The plant alkaloid tetrandrine inhibits metastasis via autophagy-dependent Wnt/β-catenin and metastatic tumor antigen 1 signaling in human liver cancer cells
Source: J Exp Clin Cancer Res. 2018 Jan 15;37:7. doi: 10.1186/s13046-018-0678-6 (PMC5769468; doi:10.1186/s13046-018-0678-6)
Supplement: Additional file 1: — Supplementary figures and figure legends. (DOCX 3775 kb) [file 13046_2018_678_MOESM1_ESM.docx]

**
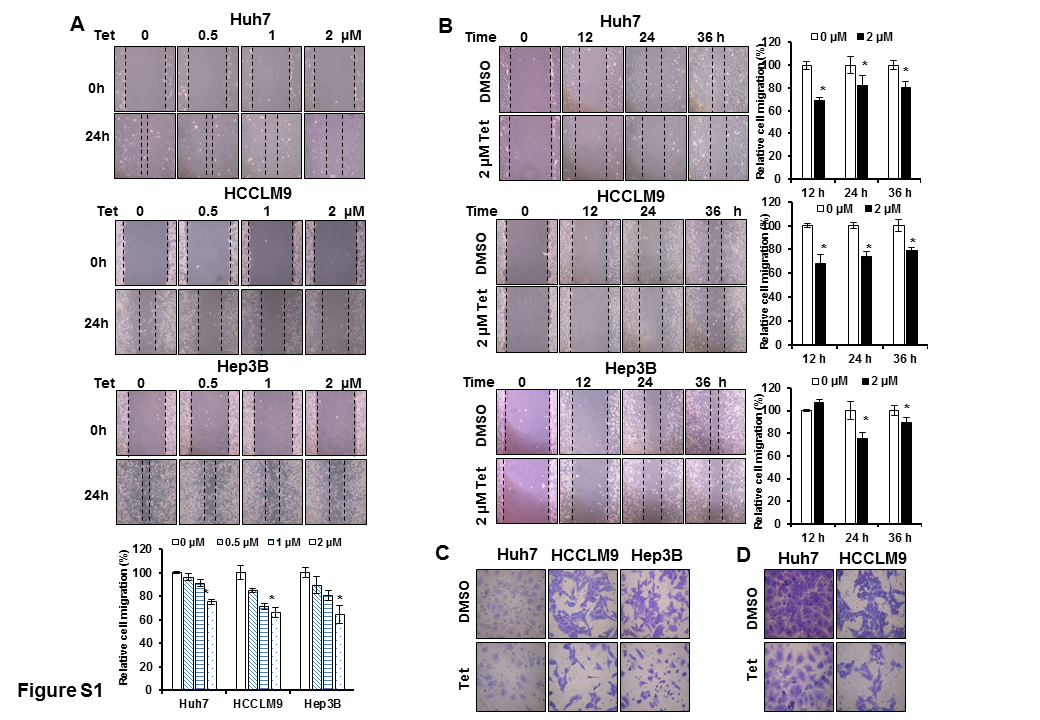
**

**Figure S1. Tetrandrine inhibits HCC cell invasion and migration**

(A) Images of wound healing in Huh7, HCCLM9 and Hep3B cells treated with indicated concentrations (0-2 µM) of tetrandrine (Tet) for 24 h. Magnification is 20X. Data are shown as the mean ± SD. *p <0.05.

(B) Images of wound healing were acquired after Huh7, HCCLM9 and Hep3B cells were treated with 2-µM tetrandrine (Tet) for indicated time intervals. Magnification is 20X. Data are shown as the mean ± SD. *p <0.05.

(C) Images of transwell migration assay and trasnwell invasion assay (D) was acquired. Magnification is 100X.

**
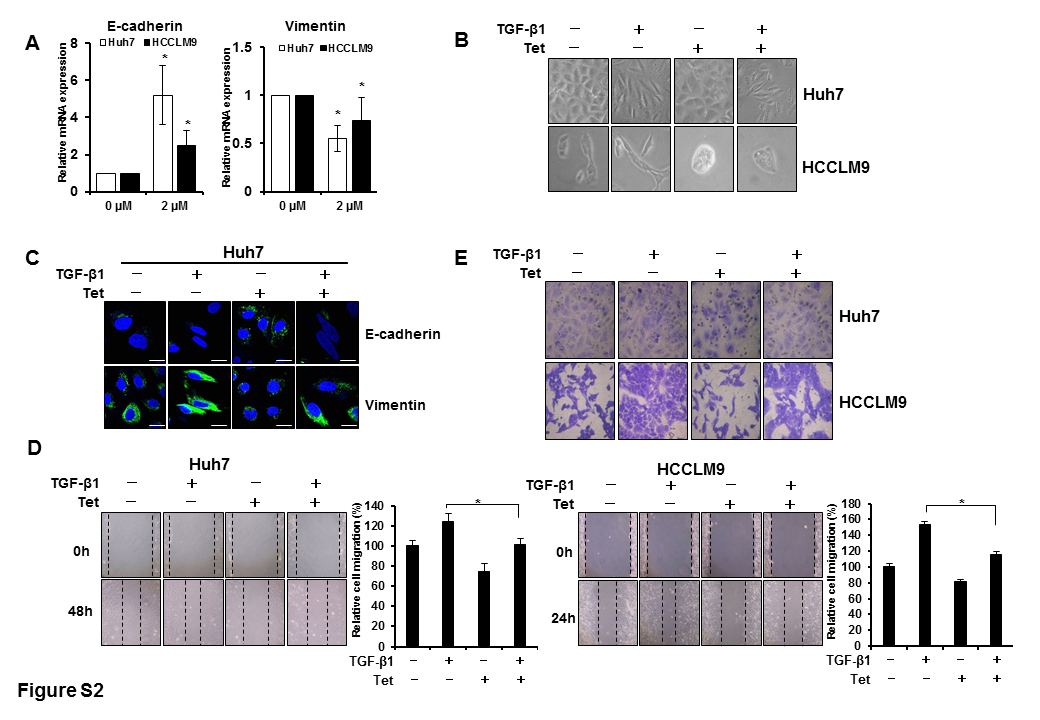
Figure S2. Tetrandrine prevented HCC cells EMT**

(A) The Huh7 and HCCLM9 cells were treated with 0 (DMSO) and 2-µM tetrandrine for 24 h, then the mRNA expression changes of the EMT-related genes E-cadherin and vimentin were determined using real-time RT-PCR. Data are represented as the mean ± SD. *p <0.05.

(B) Huh7 and HCCLM9 cells were treated with 2-µM tetrandrine (Tet) and TGF-β1 (5 ng/mL) for 72 h, control cells were only treated with DMSO. EMT was examined by assessing the cell morphological changes.

(C) Huh7 cells were treated with 2-µM tetrandrine (Tet) and TGF-β1 (5 ng/mL) for 72 h, and control cells were only treated with DMSO. E-cadherin and vimentin expression were examined by immunofluorescence staining. Scale bars: 20 µm.

(D) The images was acquired from a wound healing assay and (E) a transwell migration assay that were performed on Huh7 and HCCLM9 cells in the presence of 2-µM of tetrandrine (Tet) and 5 ng/mL of TGFβ-1 for the indicated time. Magnification is 20X (D) and 100X (E).

**
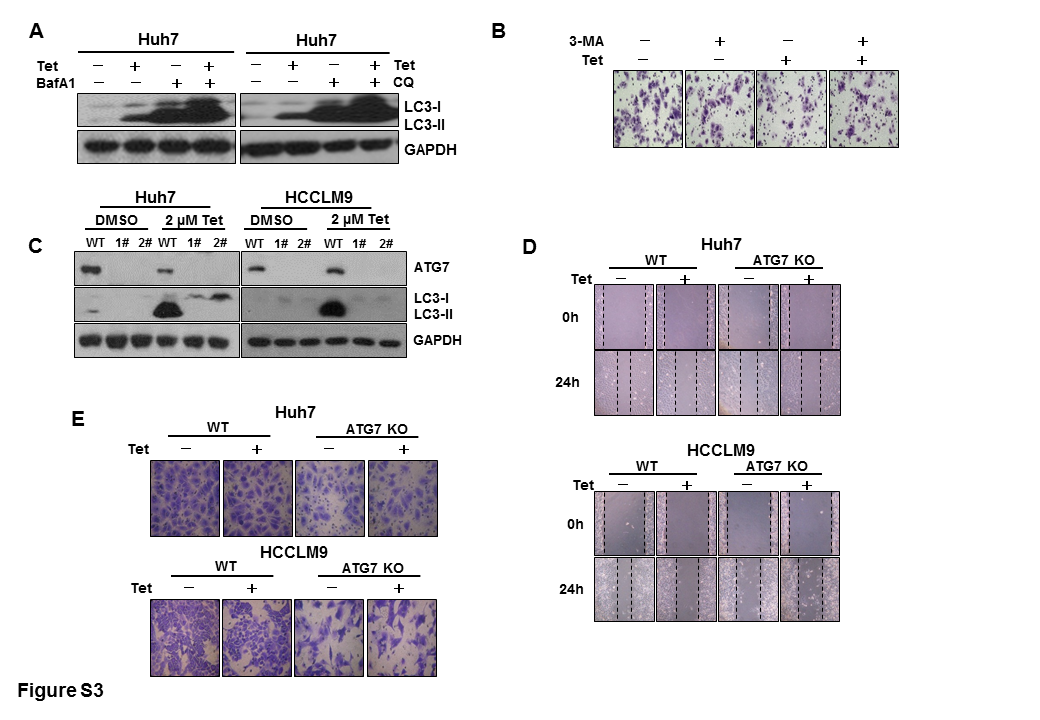
Figure S3. Tetrandrine-inhibited HCC cell migration is associated with autophagy**

(A) Huh7 cells were treated with 2-µM tetrandrine (Tet) and 20-µM CQ (Chloroquine) or 50-nM Bafilomycin (BafA1) for 24 h. Western blot analysis of LC3 and GAPDH level.

(B) A transwell migration assay was conducted on Huh7 cells in the presence of 2-µM tetrandrine (Tet) and/ or 3-MA for 24 h.

(C) Western blot analysis of ATG7 and LC3 levels in ATG7-deficient Huh7 and HCCLM9 cells that were established by using the CRISPR/Cas9 system.

(D) A wound healing assay and (E) a transwell migration assay were performed on WT and ATG7-deficient Huh7 and HCCLM9 cells in the presence of 2-µM of tetrandrine (Tet) for 24 h. Magnification is 20X (D) and 100X (E).


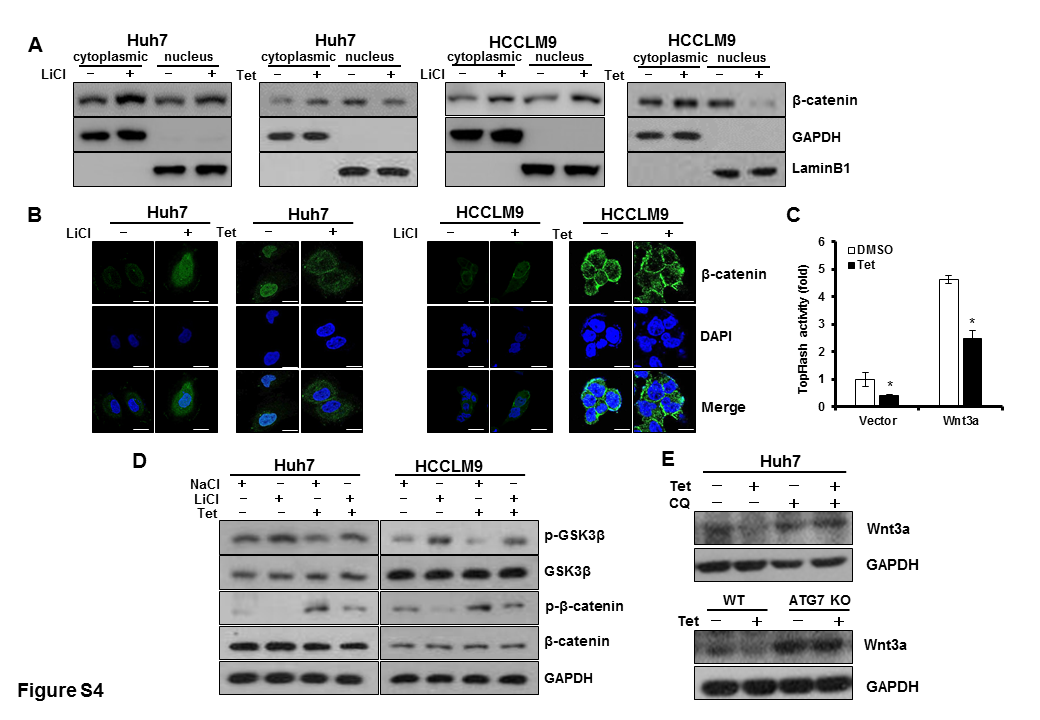


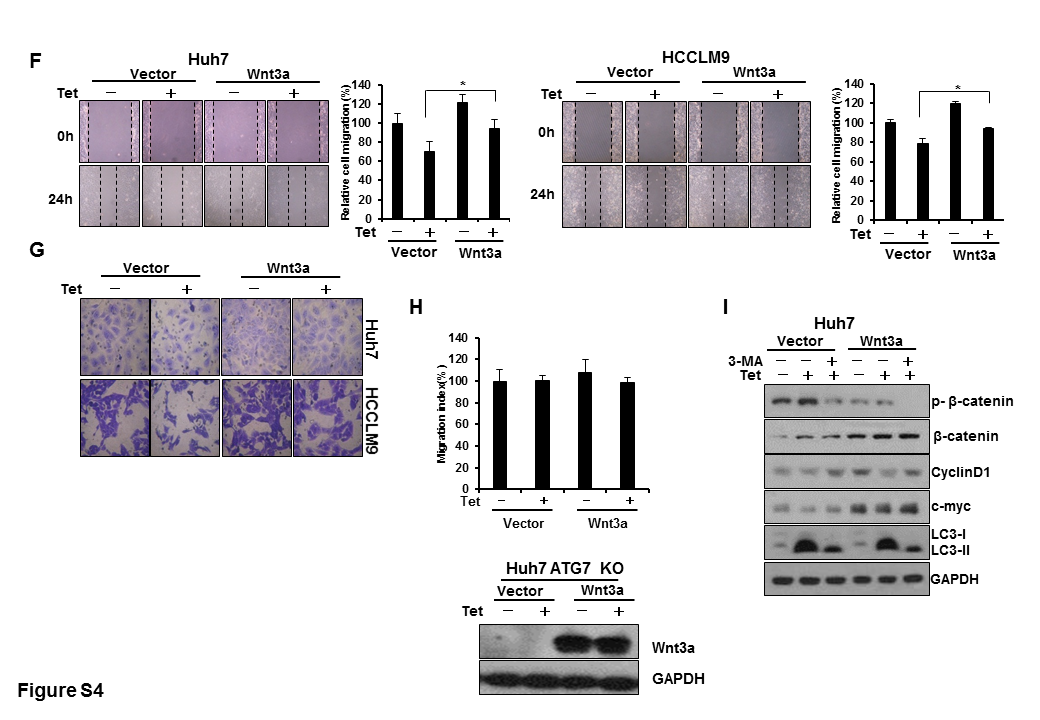


**Figure S4. Autophagy-dependent Wnt/β-catenin pathway was involved in tetrandrine- inhibition of HCC cell EMT**

(A) Western blot analysis to detect cytoplasmic and nuclear β-catenin of Huh7 and HCCLM9 cells after treatment with DMSO or 2-µM tetrandrine (Tet) for 24 h. LiCl treatment as a positive control. GAPDH and Lamin B1 was used as a cytoplasmic and nuclear protein loading control.

(B) Immunofluorescence staining to detect cellular localization of β-catenin. LiCl treatment as a positive control. Scale bars: 20 µm.

(C) Huh7-Vector and Huh7-Wnt3a overexpression cells were cotransfected with 500 ng TopFlash/FopFlash luciferase reporter and 5 ng pRL-TK plasmids (expressing *Renilla* luciferase). 48 h posttransfection, the cells were pretreated with 3-MA (3 mM) for 1 h, followed by treated with DMSO or 2 µM tetrandrine (Tet) for 4 h. subsequently lysates were prepared. The Top/Fop Flash activity was measured by the dual luciferase assay system according to the instructions. Data were presents as mean ± SD. *p <0.05.

(D) Huh7 and HCCLM9 cells were pretreated with 25-mM LiCl for 1 h, and subsequently treated in combination with DMSO or 2-µM tetrandrine (Tet) for 24 h. NaCl was used as a negative control. p-GSK3β and total GSK3β, p-β-catenin, total β-catenin protein expression was detected by western blotting.

(E) Huh7 cells were treated with 2-µM tetrandrine and 20-µM CQ (Chloroquine) for 24 h. In addition, Huh7 WT and ATG7 KO cells were treated with 2-µM tetrandrine for 24 h. Then Wnt3a expression was detected by western blotting. GAPDH served as a loading control.

(F) The images of a wound healing assay and (G) a transwell migration assay that were performed on indicated cells in presence of 2-µM of tetrandrine (Tet) for 24 h. Magnification is 20X (F) and 100X (G). Data are shown as the mean ± SD. *p <0.05.

(H) Overexpression of Wnt3a have no statistical differences in promote cell migration in ATG7 KO Huh7 cells compare with vector cells. Huh7 ATG7 KO cells were transfected with empty vector or Wnt3a plasmid, migration ability was detected by transwell migration assay. Western blot analysis of Wnt3a and GAPDH expression. Data are represented as the mean ± SD.

(I) Huh7 cells transfected with or without Wnt3a in the absence or presence of 3-mM 3-MA for 1 h were subsequently treated with 2-µM tetrandrine for 24 h. All cell lysates were subjected to western blotting to detect p-β-catenin, total β-catenin, CyclinD1, c-myc and LC3 expression. GAPDH served as a loading control.

**
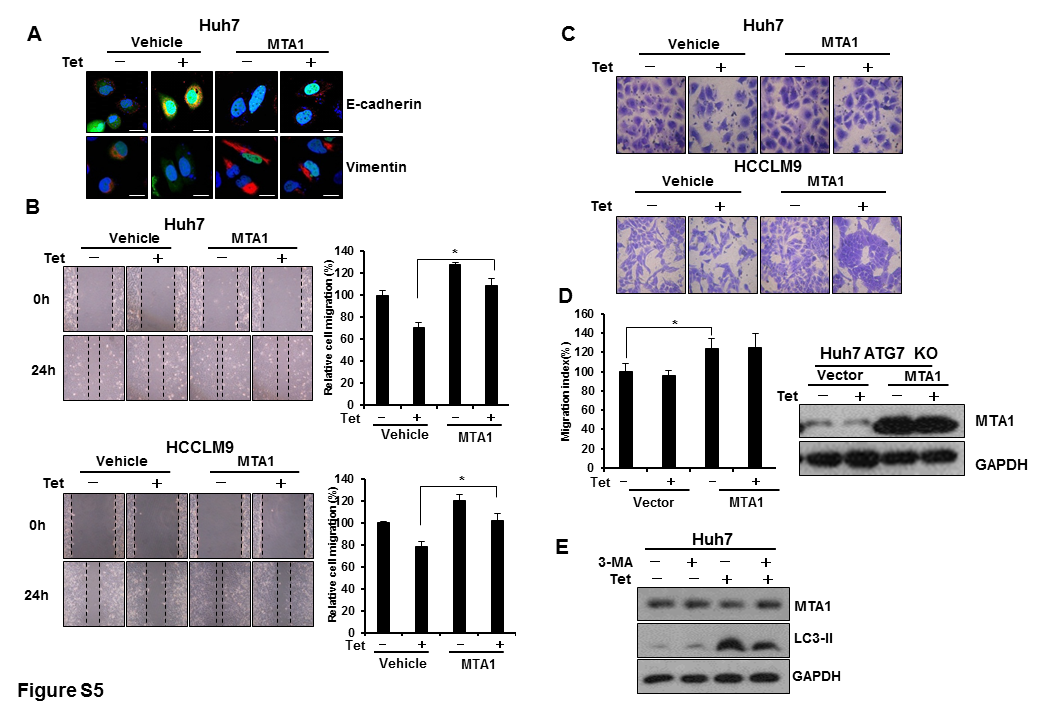
**

**Figure S5. Tetrandrine inhibited HCC migration partly through MTA1**

(A) Huh7 cells were transfected with empty vector or MTA1 overexpressing plasmid for 36 h, then cells were treated with or without 2-µM tetrandrine (Tet) for 24 h. E-cadherin and vimentin levels were detected by immunofluorescence staining. Scale bars: 20 µm.

(B) Huh7 and HCCLM9 cells were seeded in a twelve-well plate, and subsequently cells were transfected with empty vector or MTA1 overexpressing plasmid for 36 h. Then, a wound healing assay and (C) a transwell migration assay were performed on indicated cells in presence of 2-µM of tetrandrine (Tet) for 24 h. The images were acquired at 20X (B) and 100X (C) magnification. Data are shown as the mean ± SD. *p <0.05.

(D) Overexpression of MTA1 in ATG7 KO Huh7 cells can promote cell migration. Huh7 ATG7 KO cells were transfected with empty vector or MTA1 plasmid, migration ability was detected by transwell migration assay. MTA1 and GAPDH protein level was analyzed by western blotting. Data are represented as the mean ± SD. *p <0.05.

(E) MTA1 partly induced autophagy-mediated degradation. Huh7 cells were pretreated with 3-MA (3 mM) for 1 h, and subsequently treated with 2-µM tetrandrine for 24 h. Western blot analysis of MTA1 and LC3 expression. GAPDH was loaded as a control.

**
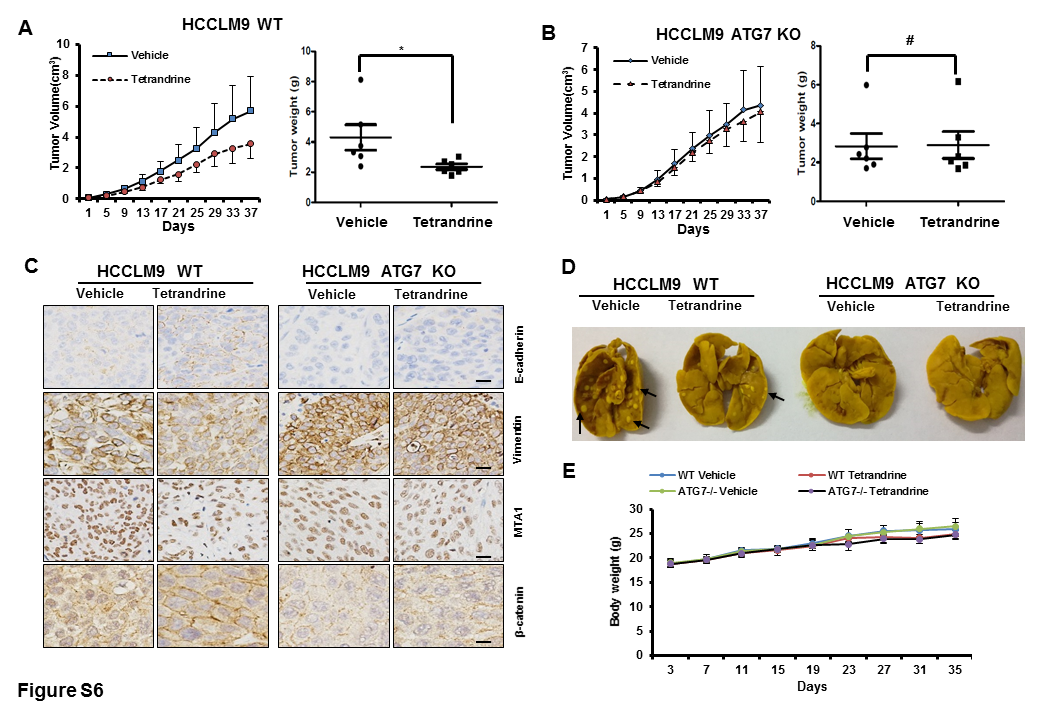
**

**Figure S6. Tetrandrine inhibits HCC metastasis *in vivo*.**

(A) Tumor volumes were measured every day in tetrandrine treatment. After mice were sacrificed, tumor weights in WT HCCLM9 or (B) ATG7-deficient HCCLM9 cell xenografts were removed and weighed. Data were presented as the mean ± SD. *p <0.05, #p>0.01.

(C) The expression level of E-cadherin, vimentin, MTA1 and β-catenin in tumor tissues was detected by immunohistochemistry staining. Scale bar: 100 µm.

(D) The lung metastatic foci in tail vein injection metastasis model was indicated by arrows.

(E) The mice body weight was measured. Data were presented as the mean ± SD.
